# Intraspecific Epitopic Variation in a Carbohydrate Antigen Exposed on the Surface of *Trichostrongylus colubriformis* Infective L3 Larvae

**DOI:** 10.1371/journal.ppat.1000597

**Published:** 2009-09-25

**Authors:** David R. Maass, Gavin B. Harrison, Warwick N. Grant, Wayne R. Hein, Charles B. Shoemaker

**Affiliations:** 1 Institute of Environmental Science and Research Ltd., Porirua, Wellington, New Zealand; 2 Hopkirk Research Institute, AgResearch Grasslands Research Centre, Palmerston North, New Zealand; 3 Genetics Department, La Trobe University, Melbourne, Australia; 4 Department of Biomedical Sciences, Tufts Cummings School of Veterinary Medicine, North Grafton, Massachusetts, United States of America; University of Pennsylvania, United States of America

## Abstract

The carbohydrate larval antigen, CarLA, is present on the exposed surface of all strongylid nematode infective L3 larvae tested, and antibodies against CarLA can promote rapid immune rejection of incoming *Trichostrongylus colubriformis* larvae in sheep. A library of ovine recombinant single chain Fv (scFv) antibody fragments, displayed on phage, was prepared from B cell mRNA of field-immune sheep. Phage displaying scFvs that bind to the surface of living exsheathed *T. colubriformis* L3 larvae were identified, and the majority of worm-binding scFvs recognized CarLA. Characterization of greater than 500 worm surface binding phage resulted in the identification of nine different anti-CarLA scFvs that recognized three distinct *T. colubriformis* CarLA epitopes based on blocking and additive ELISA. All anti-CarLA scFvs were specific to the *T. colubriformis* species of nematode. Each of the three scFv epitope classes displayed identical Western blot recognition patterns and recognized the exposed surface of living *T. colubriformis* exsheathed L3 larvae. Surprisingly, each of the anti-CarLA scFvs was able to bind to only a subset of worms. Double-labelling indirect immunofluorescence revealed that the three classes of anti-CarLA scFvs recognize distinct, non-overlapping, *T. colubriformis* sub-populations. These results demonstrate that individual *T. colubriformis* L3 larvae display only one of at least three distinct antigenic forms of CarLA on their surface at any given time, and suggest that antigenic variation within CarLA is likely a mechanism of immune evasion in strongylid nematodes.

## Author Summary

Strongylid nematode worm parasites currently infect hundreds of millions of people, and most farmed animals, causing enormous morbidity and economic loss. These parasites commonly produce chronic gastrointestinal infections that are highly refractory to immune clearance mechanisms. Mucosal antibodies against a carbohydrate surface antigen (CarLA) can cause rapid expulsion of incoming larval nematodes. Sheep develop strong anti-strongylid immunity following long-term grazing on contaminated pasture. From these sheep, we identified and characterized recombinant antibodies that recognize CarLA on living L3 stage infective larvae of the strongylid parasite, *Trichostrongylus colubriformis*. The selected antibodies are specific only to larvae of the *T. colubriformis* species and, surprisingly, recognize only a subset of these worms. Three different anti-CarLA antibody classes were found and each recognizes different, non-overlapping subsets of worms which, together, comprise virtually the entire population. These results are the first demonstration of “intraspecific epitopic variation” within strongylid nematodes and suggest that these parasites have a mechanism that permits the surface presentation of at least three different antigenic forms of CarLA to avoid immune clearance.

## Introduction

A wide variety of parasitic nematodes are capable of establishing long term chronic infections in mammals, including those that penetrate tissues and those that reside only in the gastrointestinal tract. Each of these nematode species must overcome a variety of host immune effector mechanisms without the ability of most microbial, protozoan and viral pathogens to rapidly replicate and thus overwhelm the effectors. Furthermore, they have a large and vulnerable surface, the cuticle and absorptive gut, through which they must interact closely with the host to acquire nutrients, sense their environment and otherwise coexist. Understanding how these nematodes remain refractory to immune assault at these host-interactive surfaces over the long periods of time spent in the host has been the subject of much study. General evidence has accumulated that at least some nematodes employ surface shedding, anti-oxidant enzymes, migration, camouflage, immunomodulation and possibly antigenic variation to evade the host immune system and establish chronic infections [1]. The lack of easily accessible genetic tools to manipulate genes in parasitic nematodes has hampered the ability of researchers to test specific hypotheses regarding the role and relative importance of different immune defence mechanisms.

Antigen switching is an important immune defence mechanism of protozoan parasites and some evidence exists that similar mechanisms may be available to nematode parasites. It is possible that the act of cuticle moulting during maturation of infective L3 to L4 and then again to the adult stage has been utilized by parasitic nematodes as a tool to change the surface antigen milieu [2] and may therefore play a role in immune evasion. Intraspecific variation has been reported for some nematode species and may provide the population diversity to allow some individuals to survive despite adaptive host immune effectors targeting the surface [3],[4],[5]. In a few examples, heterogeneity was reported in surface epitope expression in *Ascaris lumbricoides* [6] and *Wuchereria bancrofti* [7], although the variation may be from differential expression of multiple antigens rather than intraspecific variations that exist within specific antigens. No examples have previously been reported of nematode surface antigens that exist in different antigenic forms on individual worms of the same species. Identification of polymorphic surface antigens may have been hindered by the technical difficulties inherent in assaying surface compositions on individual worms, the paucity of clonal antibodies available to nematode parasite surface antigens, the use of relatively inbred parasite lines as models and the lack of genetic tools for studying these organisms.

The generation of a vaccine against gastro-intestinal nematodes has been the focus of numerous studies over the last thirty years. Various hurdles must be overcome before the realization of a commercial vaccine is attained; one of which is selecting the appropriate target nematode antigen(s) [8]. Clearly, the identification of promising vaccine targets benefits from better understanding of the host immune effectors that are involved in protective immunity and the mechanisms deployed by the parasites to evade these effectors. We previously reported identification of a carbohydrate larval antigen (CarLA) that is found on the surface of infectious L3 stage strongylid nematodes [9]. Levels of anti-CarLA mucosal antibodies strongly correlate with rapid immune rejection of infecting *Trichostrongylus colubriformis* [10] and purified anti-CarLA antibodies are able to elicit the rapid rejection of *T. colubriformis* L3 infection in naïve sheep [10],[11]. Here we demonstrate intraspecific variation in the presentation of CarLA epitopes on the surface of individual *T. colubriformis* L3 worms. This is the first example of epitopic variation within a single nematode surface antigen and further supports the potential of CarLA as a vaccine target of strongylid parasites.

## Materials and Methods

### Reagents

Bacterial strains, antibodies, plasmids and *T. colubriformis* L3 larvae are as described previously [12]. The vector AB6-7-myc was modified from AB6-7 [12] in which the E-Tag fusion tag was replaced by the c-myc tag. The antibody anti-c-myc-TRITC was obtained from Santa Cruz Biotechnology.

### Nematode extracts

Nematode extracts used for Western blotting were prepared by boiling intact, exsheathed larvae in SDS-PAGE sample buffer for 5 min. Insoluble material was pelleted by centrifugation at 600×g for 5 min and the supernatant was used for SDS-PAGE.

### Library construction

The ovine single chain antibody library was constructed as described previously [12]. Ovine immunoglobulin V_H_, V_λ_ and V_κ_ regions were amplified by PCR utilizing cDNA obtained from the abomasal and mesenteric lymph nodes of helminth infected sheep and the heavy and light chain variable region DNA fragments were cloned sequentially into the phage display vector HQ2-2 [12]. The total number of primary independent clones from both libraries was 1.9×10^8^. Testing of a random sampling of clones demonstrated that >95% had inserts of the appropriate size with unique BstN1 restriction patterns (data not shown).

### Library screening

Selection was carried out by “panning” of scFv-displayed phage libraries for phage that bind to exsheathed living L3 nematodes as described previously [12]. Panning on exsheathed L3 nematodes was performed by adding phage to ∼10,000 larvae that had been blocked with 4% milk powder in PBS (MPBS) at 37°C for 2 h. A 1 ml suspension of phage in MPBS was prepared containing 1.0×10^12^ colony forming units (CFU). After incubation of the phage with the worms at room temperature on a turntable for 90 min, the worms were washed 10 times with PBS containing 0.1% Tween 20 (PBST; Sigma) and 10 times with PBS by pelleting in a microfuge at 600×*g* for 2 min with unbound phage removed from the worm pellets by aspiration. The bound phage was eluted by directly adding 10 ml of log phase TG1 bacteria and incubating at room temperature for 30 min. The infected TG1 cells were collected by centrifugation, resuspended in 2 ml of 2xYT and plated onto plates containing 2xYT-AG agar for overnight incubation at 30°C. Phage were prepared from the recovered colonies as described above. Second and third round panning were performed as above except the number of washing steps before elution was increased to 20 times with PBST and PBS for the second round and 30 times with PBST and PBS for the third round.

### Production of soluble single-chain Fv domain antibodies (scFvs)

Soluble scFvs were prepared as described previously [12]. Briefly, the scFv coding DNA was ligated into the expression vectors AB6-7 or AB6-7-myc. Transformed bacterial colonies were induced with the addition of 0.2% L-arabinose (Sigma) and incubation was continued at 28°C for a further 4 hr. Soluble recombinant protein was purified from cells lysed by sonication using Ni-NTA (Qiagen) according to the manufacturer's protocol. Purity was assessed by Coomassie Blue staining of SDS-PAGE and protein concentration determined by BCA (Pierce).

### Immunoblotting

Soluble nematode extract was resolved on 12% SDS-PAGE and transferred to Immobilon-P membrane (Millipore). The membranes were blocked in 4% MPBS at room temperature for 60 min before the addition of 5 ug/ml purified scFv and incubated at room temperature for 2 hr. The membrane was washed ten times with PBST and ten times with PBS and anti-E-tag/HRP diluted at 1∶8000 in 4% MPBS was added. The membrane was incubated and washed as above and binding detected with 3-amino-9-ethyl carbazole (Sigma).

### Characterization of single-chain antibodies

#### Sequencing and analysis

Heavy and light chain sequences were determined by sequencing within the vector HQ2-2 using the sequencing primers, HQ2-2.for, GATAACAATTTCACACAAGG and HQ2-2.rev, TGAATTTTCTGTATGAGG and within the vector AB67 using the sequencing primers AraHU, ACTGTTTCTCCATACCC and AraRHU, CATCCGCCAAAACAGCC. All sequencing reactions were performed by the Waikato DNA sequencing facility and analysis performed using the suite of programs contained with Vector NTI (Invitrogen).

#### ELISA

Checkerboard titration was used to determine the optimal conditions for dilution of soluble nematode extract use for coating and dilution of scFvs for detection in the additive and blocking ELISA [13],[14]. Nunc Maxisorb plates were coated overnight at 4°C with serial dilutions of soluble nematode extract in PBS, and then blocked with 4% MPBS. After washing three times with PBS, serially diluted soluble scFvs was added and incubated at room temperature for 90 min to determine saturation curves. To detect bound scFv, an anti-E-Tag/HRP antibody (Amersham) was added at 1∶8000 dilution in 4% MPBS for 90 min at room temperature. The wells were washed and developed with 3,3′,5,5′-tetramethyl benzidine (Applichem). The optimal dilution of the antigen preparation to coat the ELISA plates was selected in which a near-maximum response was obtained.

#### Blocking ELISA

Elisa plates were prepared with soluble nematode extract as described above and a potentially blocking scFv was added at saturating concentrations (three times maximum response as determined above) and incubated for 60 min at room temperature. PBS was added to control wells containing no scFv. Serial dilutions of bacterial supernatants containing phage were then added for 90 min incubation at room temperature. Plates were then washed three times with PBST and three times with PBS. To detect bound phage, an anti-M13/HRP antibody (Amersham) was added at 1∶8000 dilution in 4% MPBS for 90 min at room temperature. The wells were washed and developed as described above. Percent binding was calculated as the OD_test_/OD_control_×100.

#### Additive ELISA

Additive ELISA was based on the method of Friguet et. al. [14] Plates were prepared as described for standard ELISA above and saturating concentrations of each scFV as determined above was added individually and in pairs. After incubation and washing as described previously, bound antibody was detected by the addition of anti-E-tag/HRP antibody diluted at 1∶8000. The additivity index (A.I.) was determine by A.I. = (2A_1+2_/A_1_+A_2_−1)×100 where A_1_, A_2_ and A_1+2_ are the absorptions reached, in the ELISA, with the first scFv alone, the second scFv alone and the two scFv together [14].

### Immunofluorescence

Immunofluorescence on exsheathed *T. colubriformis* L3 larvae was performed by adding 1 ug/ml soluble scFv singly or in combination to 1000 larvae that were blocked with 4% MPBS at 37°C for 1 h. After incubation of the scFv with the worms at room temperature on a turntable, the worms were washed 5 times with PBST and 5 times with PBS by pelleting worms by centrifugation in a microfuge for 2 min. Anti-c-myc antibody (Santa Cruz Biotechnology) was conjugated with R-phycoerythrin using the Protein-Protein Crosslinking Kit (Invitrogen), while anti-E-Tag antibody (Pharmacia) was conjugated using the AlexaFluor 488 Protein Labelling Kit (Invitrogen). Anti-E-Tag/FITC and/or anti-c-myc antibody were added at a dilution of 1/500 in 4% MPBS and incubated and washed as described above. Labelled larvae were visualized by Leitz fluorescence microscopy.

## Results

### Selection of scFvs that bind to living *T. colubriformis* L3 larvae

Two-year-old Romney sheep were raised on pasture known to contain *T. colubriformis*, *Haemonchus contortus* and *Teladorsagia circumcincta* and were shown to be refractory to infection. DNA encoding the mucosal antibody variable-region repertoire from these field immune sheep was amplified by PCR and used to generate a single chain Fv (scFv) library displayed on the surface of M13 phage. Phage-displayed scFvs that recognize the surface of living *T. colubriformis* L3 larvae were obtained by incubating the library with the worms, washing and then eluting bound phage. The eluted phage were amplified to initiate a subsequent panning cycle. Phage recovered following three cycles of live L3 worm panning were screened by ELISA for recognition of a soluble extract of boiled L3 larvae previously shown to be highly enriched for a carbohydrate surface antigen of *T. colubriformis* L3 larvae termed CarLA [12]. Greater than 80% of the screened phage displayed a positive ELISA signal. Over five hundred ELISA positive clones were subjected to DNA fingerprinting and yielded 35 unique scFv clones (data not shown).

### Western analysis of scFvs

**Figure 1:**
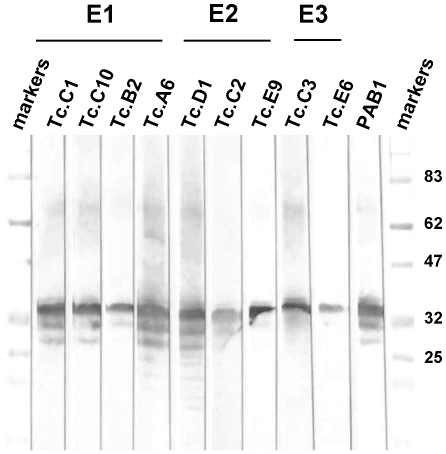
Western blots of nine selected ovine scFvs for recognition of antigens in *T. colubriformis* L3 larval extracts. Soluble nematode L3 larval extract from *T. colubriformis* was separated on 11% SDS-PAGE and probed with the specified scFvs or PAB-1 (anti-CarLA mAb). The indicated scFvs were incubated with the blots and bound scFv detected using an anti-E-tag/HRP conjugated mAb. scFv members recognizing the three epitope groups, E1, E2 and E3, are indicated at the top.

A scFv clone representing each of the 35 unique DNA fingerprint classes was subcloned in the *E. coli* expression vector AB6-7 and soluble recombinant protein was produced. Each scFv was expressed with a short carboxyl terminal peptide epitopic tag, E-tag, to facilitate immunologic detection. Nine of the 35 unique scFv ELISA positive clones (representing 70% of the ELISA positive clones) produced the same recognition pattern in Western blots of larval extracts, a major band migrating at 35 kDa atop a diminishing ladder of lower MW species (Figure 1). This banding pattern is highly characteristic of *T. colubriformis* CarLA [9] and virtually identical to the pattern recognized by PAB-1 [10], an anti-CarLA mAb (Figure 1).

### Epitope characterization for the nine unique anti-CarLA scFvs

**Figure 2:**
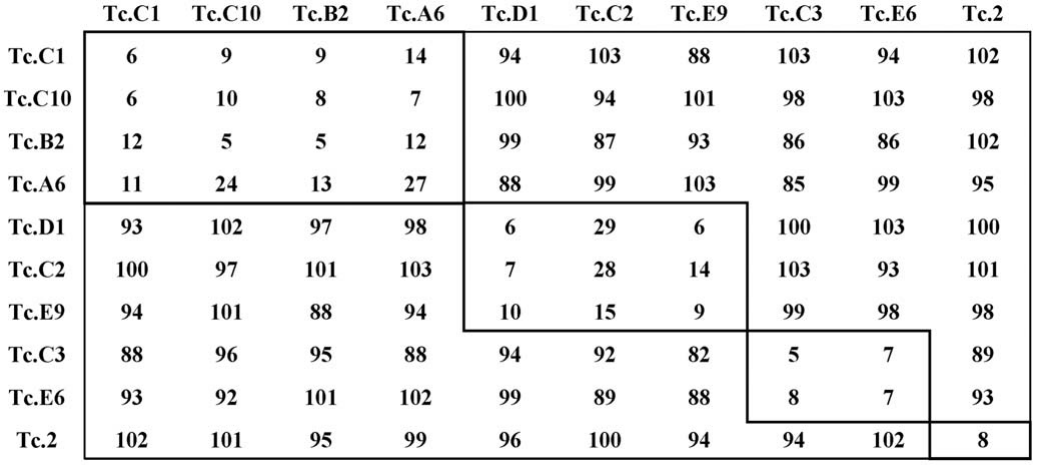
Blocking ELISA for epitope analysis of the anti-*T. colubriformis* CarLA scFvs. Phage displaying scFv (top row) was added to wells coated with *T. colubriformis* extract that had been pre-treated with soluble expressed scFv (first column). Blocking by the soluble scFvs is reflected by substantially reduced signals produced by the phage-displayed scFvs. Values represent percent binding compared to PBS controls. Wells displaying greater than 70% signal reduction (blocking) are boxed. The assay was performed in duplicate on at least three separate occasions.

Blocking and additive ELISA methods were employed to establish epitope grouping among the anti-CarLA scFvs. Figure 2 shows the results of blocking ELISAs in which each of the nine purified recombinant anti-CarLA scFvs, or a control anti-sheath scFv, Tc.2 [12], were tested for the ability to block the binding of the phage displayed scFvs to extracts containing CarLA. This analysis identified three distinct epitope groups. Group E1 was comprised of Tc.A6, Tc.B2 Tc.C1and Tc.C10; group E2 included Tc.C2 Tc.D1 and Tc.E9; and group E3 contained Tc.C3 and Tc.E6. As expected, Tc.2 phage binding, which binds to a sheath antigen that is also present in the extracts, was not inhibited by any of the soluble scFvs. These results were independently confirmed using additive ELISA methods [14] (Figure S1). Two representatives of scFvs from group E1, E2 and E3 were demonstrated by ELISA to recognize CarLA that was purified by immunoaffinity with the anti-CarLA mAb, PAB-1 [9]. The scFvs, Tc.1 and Tc.2 which recognize other surface antigens in crude extracts [12], did not react with the PAB1 purified CarLA thus confirming that the target of the nine scFvs is CarLA (Table S1).

**Figure 3:**
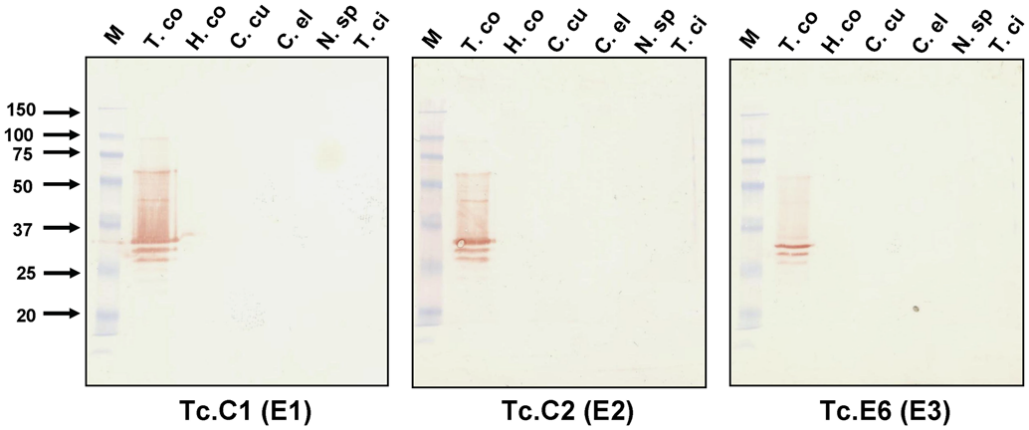
Species specificity of anti-CarLA scFvs representing three different epitope classes, E1, E2 and E3. Western blots were performed containing hot water extracts of L3 stage larvae prepared from six different species of nematode. Species used were *Trichostrongylus colubriformis* (T. co), *Haemonchus contortus* (H. co), *Cooperia cur*ticei (C. cu), *Caenorhabditis elegans* (C. el), *Nematodirus spatiger* (N. sp) and *Teladorsagia circumcincta* (T. ci). The indicated scFvs were incubated with the blots and bound scFv was detected using an anti-E-tag/HRP conjugated mAb. Markers (M) indicate the molecular weight mobility shown on left.

In previous work, we found that anti-CarLA scFvs that bind to epitopes present on the host-interactive surface of nematodes were species-specific [12]. To determine if species-specificity was also a property of the new anti-CarLA scFvs, one scFv member of each anti-CarLA epitope class was tested for recognition of CarLA from a variety of nematode species. As seen in Figure 3, each of the anti-CarLA scFvs recognize CarLA only within extracts of *T. colubriformis*, the species used to select these scFvs from the phage-display library (see above). This specificity was not observed when blots containing the same extracts were performed using the anti-CarLA mAb, PAB-1 [10] or an scFv recognizing a sheath antigen common to strongylid L3s (scFv Tc.2 [12], Figure S2). PAB-1 recognized CarLA at a non-surface epitope in each of the strongylid extracts tested in Figure 3 (see reference [10]). Despite substantial efforts with *T. colubriformis* (this paper) and several other species ([12] and unpublished data), we have yet to find a host-exposed CarLA epitope that is not species-specific.

### Coding sequences of the scFvs

The heavy and light chain variable region of each CarLA specific fingerprint class member was sequenced (Genbank accession # FJ670532-41) and compared to each other and to published scFv sequences. As expected, all of the scFv heavy and light chains were found to be most homologous to ovine immunoglobulin sequences in a Genbank search. The alignment of the translated sequences is shown in Supporting Figure S3, and demonstrates the typical, extensive homology throughout the framework domains and variability within the complementarity determining regions (CDRs) [15]. There were examples of different scFv clones containing virtually identical heavy chains (see Tc.C1 and Tc.B2 or Tc.C2 and Tc.E9) but different light chains. Also there were examples of different scFv clones with virtually identical light chains (see Tc.C2 and Tc.D1 or Tc.B2 and Tc.C10) but different heavy chains. This suggests that the heavy chain and the light chain can both contribute substantially to CarLA epitope specificity and that significant promiscuity of heavy and light chain combinations is tolerated without loss of specificity.

### Anti-CarLA scFvs recognize only a subset of living L3 nematodes

**Figure 4:**
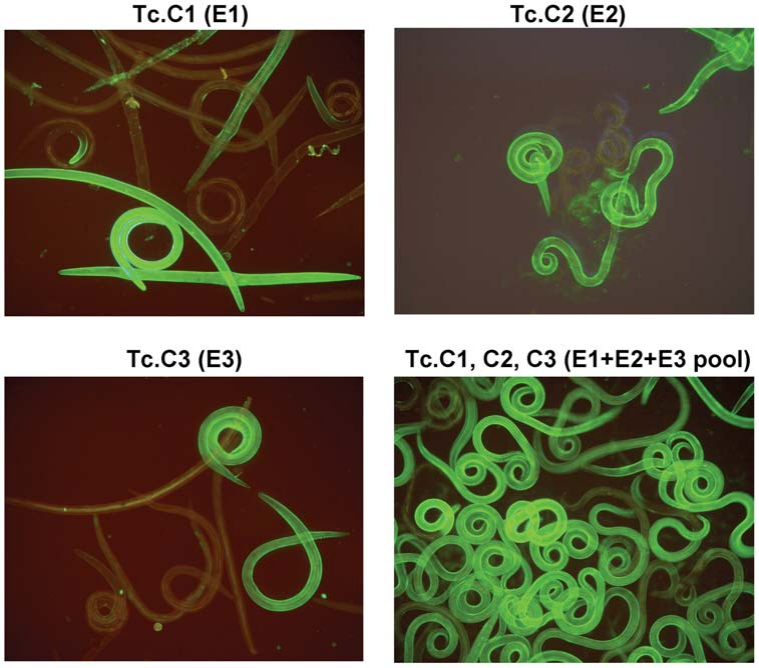
Immunofluorescence of living *T. colubriformis* L3 larvae probed with selected anti-CarLA scFvs individually or in combination. Live *T. colubriformis* exsheathed L3 larvae were probed with Tc.C1 scFv alone, Tc.C2 scFv alone, Tc.C3 scFv alone, or all three in combination as indicated. Bound scFv was stained with anti-E-tag/FITC conjugated mAb and detected by fluorescence microscopy.

The bacterial expressed scFvs were incubated individually with living *T. colubriformis* L3 larvae and bound scFv was visualized with anti-E-Tag/FITC. All of the nine anti-CarLA scFvs bound to the surface of the exsheathed *T. colubriformis* nematode although, surprisingly, none were able to stain all the larvae in the population. As seen in Figure 4, members of epitope E1 and E2 groups bound to approximately 40–50% of the population whereas members of epitope group E3 bound about 10–20% of the larvae. When the scFvs in the different epitope groups were incubated with exsheathed *T. colubriformis* L3 larvae in combinations, the percentage of the population exhibiting binding increased proportionally to the amount that each individual scFv epitope group member were able to bind singularly. As seen in Figure 4, greater than 95% of the larvae in the population were stained when scFvs for all three anti-CarLA epitope classes, E1, E2 and E3, were combined. Although completely unstained worms were observed occasionally, our results indicate that these three classes of CarLA represent the vast majority of all *T. colubriformis* L3 larvae in our worm population.

### Different anti-CarLA scFvs recognize different L3 nematode subsets

**Figure 5:**
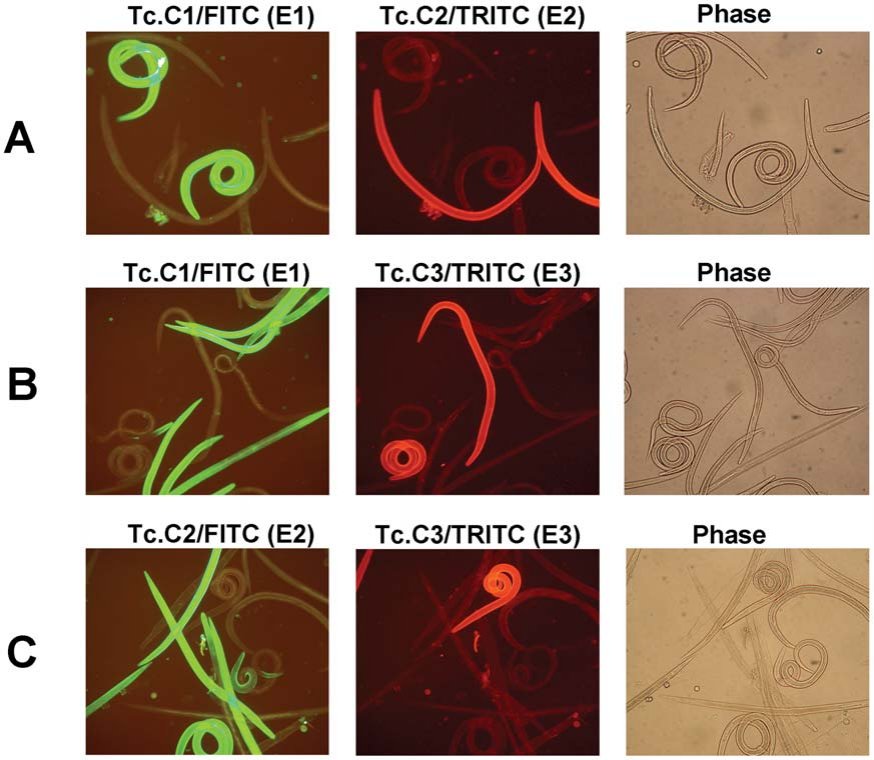
Double-staining immunofluorescence of living *T. colubriformis* L3 larvae probed with two anti-CarLA scFvs in various epitope group combinations. Live *T. colubriformis* exsheathed L3 larvae were incubated with pools of two anti-CarLA scFvs, each recognizing a different epitope and fused to either the E-tag or the c-myc epitopic tag. After washing, bound scFv was stained with both anti-E-tag/FITC mAb and anti-c-myc/TRITC mAb and detected by fluorescence microscopy using filters optimized for FITC (green) or TRITC (red) detection. Within each group (A–C) the same fields were visualized for FITC (left) and TRITC (center) or by phase contrast (right). A. The scFv pool was Tc.C1/E-Tag and Tc.C2/c-myc. B. The scFv pool was Tc.C1/E-Tag and Tc.C3/c-myc. C. The scFv pool was Tc.C2/E-Tag and Tc.C3/c-myc. The epitope group of the anti-CarLA scFvs are indicated in parentheses.

To permit double-labelling immunofluorescence with different scFvs, one scFv member of each of the three epitope groups was subcloned into an expression vector such that its coding DNA was fused in frame to the c-myc epitope in place of E-tag. Using the same scFvs with different epitopic tags did not change their specificity for a specific subset of larval worms and the same subset of worms became stained (Figure S4). Various pairs of scFvs with different CarLA epitope recognition, and having different fusion tags for detection, were incubated together with exsheathed *T. colubriformis* L3 larvae and the bound scFvs were detected with either anti-E-Tag/FITC or anti-c-myc/TRITC. In each combination examined there was a distinct subset of worms staining with each scFv. It can be seen in Figure 5 that when Tc.C1 and Tc.C2 were incubated together there is observed a subset of nematodes which bound Tc.C1 and another subset of nematodes which bound Tc.C2. In addition there was the presence of worms that bound neither Tc.C1 nor Tc.C2. Strikingly, there was no evidence of any worms which bound both Tc.C1 and Tc.C2. The distinct binding pattern observed is not caused by competition by the other scFv as the subset binding patterns were identical when the combinations of scFv were incubated with worms sequentially or as a pool (Figure S5). The existence of distinct worm subsets recognized by scFvs of different epitope binding types was maintained using the other pairs of scFvs, Tc.C1 and Tc.C3. and Tc.C2 and Tc.C3 (Figure 5). Additional examples of double staining by pairs of these and other anti-CarLA scFvs having different epitope specificity are shown in Supporting Figures S6 and S7.

## Discussion

Previous studies identified the strongylid L3 stage-specific carbohydrate surface antigen, CarLA, as a major target of mucosal antibodies in infected sheep [12] and demonstrated that purified anti-CarLA antibodies are sufficient to cause rapid rejection of incoming *T. colubriformis* L3 larvae. Although the role of CarLA in the infection process is unclear, its localization at the living nematode L3 surface and its susceptibility to antibody-mediated worm clearance suggest that a mechanism which permitted CarLA to avoid host immune recognition would be important for the parasite. We propose that the epitopic variation we report here may provide such a mechanism.

We utilized an ovine scFv library generated from sheep naturally immune to *T. colubriformis* infection, thus expecting that the antibody repertoire represented in this scFv library would be a rich source for antibodies directed at the surface of the nematode. This proved correct, as we were able to select many different scFvs from this library that recognize L3 stage surface epitopes on three different strongylid parasite species [12]. The majority of these scFvs recognize the carbohydrate larval antigen, CarLA.

Interestingly, all of the anti-CarLA scFvs we found that recognized the surface of living worms were able to recognize CarLA only from one strongylid species. Furthermore, mucosal antibodies obtained from monospecific *T. colubriformis* immune sheep do not cross-react with CarLA from other nematode species (Figure 3). This species specificity was not observed for scFvs recognizing the other two major L3 antigens exposed on the living worm surface [12]. All scFvs obtained to these antigens cross-react with similar antigens in each of the other strongylids tested [12]. Through characterization of a monoclonal antibody, PAB-1, we know that at least one CarLA epitope, which is not exposed at the worm surface, is common to CarLA from most or all strongylid parasites. PAB-1 was obtained from mice immunized against partially purified CarLA extracts and is able to recognize CarLA on Western blots from a wide variety of strongylid L3 stage larvae [9]. Although PAB-1 does not recognize the surface of living worms, it will bind to the surface of dead worms following a lethal heat treatment [10]. These observations show that while some epitopic regions within CarLA are highly conserved, only species-specific epitopes are accessible to host antibodies on living worms, and strongly suggest an evolutionary selection to reduce immune recognition of CarLA.

In this paper, through characterization of individual anti-CarLA scFvs, we further show that CarLA displays intraspecific variation. Intraspecific epitopic variation within *T. colubriformis* CarLA was proven through the identification of scFvs recognizing distinct CarLA epitopes exposed on the L3 surface and the demonstration that each scFv recognizes a different, non-overlapping group of L3 worms. Thus we have shown that *T. colubriformis* CarLA can be exposed on the L3 surface in at least three different epitopic forms, but only one of these forms is displayed on each individual worm. The use of field immune animals as the library source argues that the three CarLA epitopes identified in this study are represented in an outbred field population. It is likely that other epitopic forms of *T. colubriformis* CarLA exist in nature as evidenced by our occasional finding of individual worms (<5%) that remained unstained by the three scFvs recognizing the three CarLA variations. This is the first clear demonstration of intraspecific epitopic variation amongst nematode surface antigens and suggests the possibility that strongylid parasites may be capable of some form of antigen switching as an immune evasion mechanism.

The fact that we did not find worms recognized by more than one anti-CarLA scFv implies, quite surprisingly, that only one CarLA epitope is generally available on any single larval worm. It is thus interesting to consider how the worm might introduce intraspecific variation within an antigen normally exposing only a single epitope. CarLA was previously reported to be a carbohydrate antigen based mostly on the nature of its resistance to chemical hydrolysis and its staining by biotin hydrazide [9]. No evidence of a CarLA protein component was found based on resistance to proteases and harsh chemical treatments and the inability of CarLA to bind various protein staining reagents [9]. CarLA was also found to be resistant to digestion by all lipases and glycosidases tested [9]. Preliminary component analysis performed recently on purified CarLA found evidence for the presence of several different neutral and amino sugars and a few fatty acids, but detected no amino acids (data not shown), indicating that CarLA may be a novel glycolipid. If so, CarLA would not be the direct product of gene expression but rather the product of an as yet unknown glycolipid biosynthetic pathway. Variation could thus not be introduced via polymorphism or changes in the expression of the gene that encodes the surface antigen such as seen in trypansomes and malaria. Since all the different epitopic forms migrate identically during SDS-PAGE (Figure 1), the epitopic differences are likely subtle alterations not due to major structural changes or chemical modifications.

Glycans typically have highly repeated structures and our results with CarLA are consistent with the concept that epitopic variants occur because of minor changes in this repeated motif. It could be that particular intra- or inter-molecular linkages differ between the epitopic groups, perhaps via the expression of different CarLA biosynthesis or modifying enzymes as has been previously shown for glycolipids in the intestine of *C. elegans* [16]. In this regard, it will be of much interest to determine whether the variation in CarLA epitopes behaves as a single Mendelian trait or is determined through the action of several genes and exhibits a more complex mode of inheritance. Availability of our anti-CarLA scFvs will permit purification of worms expressing a particular epitopic variation of CarLA for re-insertion into animals so we can assess the epitopic nature of the resulting progeny. It is also of interest to determine whether the variation we have observed is the product of underlying genetic polymorphism (i.e. each epitope is the product of a single genotype), or whether a single genotype can generate more than one epitope via differential expression of a suite of “CarLA biosynthesis” enzymes.

It is intriguing that each of the different anti-CarLA scFvs recognizing the mucosal browsers, *T. colubriformis* and *T. circumcincta*, bind to only a subset of the population, while anti-CarLA scFvs recognizing the blood feeder, *H. contortus*, bind to the total population of worms ([12] and this study). This may simply be the result of our sampling a less heterogeneous population of *H. contortus* within our field site. Alternatively, the immune response against blood feeding worms may differ in some way that has reduced the evolutionary pressure for *H. contortus* to display surface antigenic variation as in the gastrointestinal browsers. Similar investigations of CarLA from other nematode species will be required to determine if intraspecific variation in this antigen is a widespread phenomenon.

CarLA is an attractive candidate as a vaccine antigen as it seems to be a major target of protective mucosal antibodies effective against *T. colubriformis* L3 [10] and antibodies binding to the surface of *T. colubriformis* L3 larvae are able to elicit rapid rejection of incoming worms [10],[11]. However, due to the diversity of CarLA epitopes that are exposed on different strongylid L3 larvae, and the intraspecific variation that exists within at least *T. colubriformis*, development of a broadly effective vaccine based on CarLA may need to induce potent mucosal antibody responses against virtually all CarLA epitopes or it will simply select for worm populations displaying other CarLA epitopes. We suggest that it was this same evolutionary pressure that has led to the current intraspecific variation within CarLA.

## Supporting Information

Table S1 The anti-*T. colubriformis* CarLA scFvs recognize immuno-purified CarLA. Plates were coated with either PAB1 purified CarLA [9], soluble nematode extract or alkaline nematode extract [12] as for blocking ELISA. Selected ovine soluble scFvs, each fused to E-tag, were incubated in the wells. Bound scFv was detected by anti-E-tag/HRP antibody as described in Methods.
(0.04 MB DOC)

Figure S1 Additive ELISA for epitope analysis of each of the nine anti-*T. colubriformis* CarLA scFvs. The additive ELISA was performed based on the method of Friguet J et. al. [14]. The plates were coated with CarLA as for the blocking ELISA. Soluble scFvs were then added to the wells individually or in pairs using a saturating dilution of each scFv (three times maximum response as determined by ELISA). Bound scFv was detected by anti-E-tag/HRP antibody as previously described. The additivity index (A.I.) was determined by A.I. = (2A_1+2_/A_1_+A_2_−1)×100 where A_1_, A_2_ and A_1+2_ are: the observed absorption signals following ELISA well development with the first scFv alone; the second scFv alone; and the two scFv together, respectively. Wells displaying an A.I. less than 25 are boxed.
(0.03 MB PDF)

Figure S2 Lack of species specificity of Tc.2 scFv recognizing a strongylid sheath antigen-loading control for Figure 2. Western blot was performed containing hot water extracts of L3 stage larvae prepared from eight different species of nematode. Species used were *Trichostrongylus colubriformis* (T. co), *Teladorsagia circumcincta* (T. ci), *Caenorhabditis elegans* (C. el), *Ostertagia ostergia* (O. os), *Cooperia curticei* (C. cu), *Parastrongyloides trichosuri* (P. tr), *Haemonchus contortus* (H. co), and *Nematodirus spatiger* (N. sp). The Tc.2 scFv [12] was incubated with the blots and bound scFv was detected using an anti-E-tag/HRP conjugated mAb. Markers (M) indicate the molecular weight mobility shown on left.
(0.13 MB PDF)

Figure S3 Sequence analysis of selected ovine scFvs. The amino acid sequences of the nine selected anti-CarLA scFvs are presented in single letter amino acid code. Alignment of the sequences was performed using DS-Gene software (Accelrys Gene 2.0). Darker shading indicates more conservation of amino acid sequence at that position.
(0.31 MB PDF)

Figure S4 Double-staining immunofluorescence of living *T. colubriformis* L3 larvae probed with the same anti-CarLA scFvs fused to different epitope tags. Live *T. colubriformis* exsheathed L3 larvae were incubated with pools containing the same anti-CarLA scFv (as indicated) fused to two different tags, E-tag or the c-myc. After washing, bound scFv was stained with both anti-E-tag/FITC mAb and anti-c-myc/TRITC mAb and detected by fluorescence microscopy using filters optimized for FITC (green) or TRITC (red) detection. Within each group the same fields were visualized for FITC (left) and TRITC (center) or by phase contrast (right).
(3.08 MB PDF)

Figure S5 Double-staining immunofluorescence of living *T. colubriformis* L3 larvae probed with two anti-CarLA scFvs using sequential or pooled scFv incubations. A. Live *T. colubriformis* exsheathed L3 larvae were incubated with anti-CarLA scFv Tc.D1, washed, and then incubated with scFv Tc.C3. B. Live *T. colubriformis* exsheathed L3 larvae were incubated with a pool containing anti-CarLA scFvs Tc.D1 and Tc.C3. After washing, bound scFv was stained with both anti-E-tag/FITC mAb and anti-c-myc/TRITC mAb and detected by fluorescence microscopy using filters optimized for FITC (green) or TRITC (red) detection. Within each group the same fields were visualized for FITC (left) and TRITC (center) or by phase contrast (right). The epitope group of the anti-CarLA scFvs are indicated in parentheses.
(2.47 MB PDF)

Figure S6 Double-staining immunofluorescence of living *T. colubriformis* L3 larvae probed with two anti-CarLA scFvs in various epitope group combinations. Live *T. colubriformis* exsheathed L3 larvae were incubated with pools of two anti-CarLA scFvs, each recognizing a different epitope and fused to either the E-tag or the c-myc epitopic tag. After washing, bound scFv was stained with both anti-E-tag/FITC mAb and anti-c-myc/TRITC mAb and detected by fluorescence microscopy using filters optimized for FITC (green) or TRITC (red) detection. Within each group (A-C) the same fields are visualized for FITC (left) and TRITC (center) or by phase contrast (right). A. The scFv pool was Tc.D1/E-Tag and Tc.A6/c-myc. B. The scFv pool was Tc.D1/E-Tag and Tc.C1/c-myc. C. The scFv pool was Tc.C1/E-Tag and Tc.D1/c-myc. The epitope group of the anti-CarLA scFvs are indicated in parentheses.
(2.34 MB PDF)

Figure S7 Double-staining immunofluorescence of living *T. colubriformis* L3 larvae probed with two anti-CarLA scFvs in various epitope group combinations. Live *T. colubriformis* exsheathed L3 larvae were incubated with pools of two anti-CarLA scFvs, each recognizing a different epitope and fused to either the E-tag or the c-myc epitopic tag. After washing, bound scFv was stained with both anti-E-tag/FITC mAb and anti-c-myc/TRITC mAb and detected by fluorescence microscopy using filters optimized for FITC (green) or TRITC (red) detection. Within each group (A–C) the same fields are visualized for FITC (left) and TRITC (center) or by phase contrast (right). A. The scFv pool was Tc.A5/E-Tag and Tc.C1/c-myc. B. The scFv pool was Tc.C3/E-Tag and Tc.C1/c-myc. C. The scFv pool was Tc.C3/E-Tag and Tc.C2/c-myc. The epitope group of the anti-CarLA scFvs are indicated in parentheses.
(2.27 MB PDF)
