# Supplementary material for: Intraspecific Epitopic Variation in a Carbohydrate Antigen Exposed on the Surface of Trichostrongylus colubriformis Infective L3 Larvae
Source: PLoS Pathog. 2009 Sep 25;5(9):e1000597. doi: 10.1371/journal.ppat.1000597 (PMC2742895; doi:10.1371/journal.ppat.1000597)
Supplement: Table S1 — The anti-T. colubriformis CarLA scFvs recognize immuno-purified CarLA. Plates were coated with either PAB1 purified CarLA [9], soluble nematode extract or alkaline nematode extract [12] as for blocking ELISA. Selected ovine soluble scFvs, each fused to E-tag, were incubated in the wells. Bound scFv was detected by anti-E-tag/HRP antibody as described in Methods. (0.04 MB DOC) [file ppat.1000597.s001.doc]

**Supporting Information**

Table S1. **The anti-*T. colubriformis* CarLA scFvs recognize immuno-purified CarLA.** Plates were coated with either PAB1 purified CarLA (9), soluble nematode extract or alkaline nematode extract (12) as for blocking ELISA. Selected ovine soluble scFvs, each fused to E-tag, were incubated in the wells. Bound scFv was detected by anti-E-tag/HRP antibody as described in Methods.

|  | **Tc.C1** | **Tc.A6** | **Tc.C2** | **Tc.D1** | **Tc.C3** | **Tc.A5** | **Tc.2** | **Tc.1** | **PAB1** |
| --- | --- | --- | --- | --- | --- | --- | --- | --- | --- |
| **PAB1-CarLA** | 0.61 | 0.78 | 0.71 | 0.67 | 0.54 | 0.4 | 0.05 | 0.06 | 0.47 |
| **soluble extract** | 1.01 | 0.87 | 0.83 | 0.88 | 0.67 | 0.58 | 0.86 | 0.57 | 0.66 |
| **alkaline extract** | 0.88 | 0.88 | 0.81 | 0.81 | 0.65 | 0.48 | 0.87 | 0.11 | 0.63 |
