# Supplementary material for: Intraspecific Epitopic Variation in a Carbohydrate Antigen Exposed on the Surface of Trichostrongylus colubriformis Infective L3 Larvae
Source: PLoS Pathog. 2009 Sep 25;5(9):e1000597. doi: 10.1371/journal.ppat.1000597 (PMC2742895; doi:10.1371/journal.ppat.1000597)
Supplement: Figure S1 — Additive ELISA for epitope analysis of each of the nine anti-T. colubriformis CarLA scFvs. The additive ELISA was performed based on the method of Friguet J et. al. [14]. The plates were coated with CarLA as for the blocking ELISA. Soluble scFvs were then added to the wells individually or in pairs using a saturating dilution of each scFv (three times maximum response as determined by ELISA). Bound scFv was detected by anti-E-tag/HRP antibody as previously described. The additivity index (A.I.) was determined by A.I. = (2A1+2/A1+A2−1)×100 where A1, A2 and A1+2 are: the observed absorption signals following ELISA well development with the first scFv alone; the second scFv alone; and the two scFv together, respectively. Wells displaying an A.I. less than 25 are boxed. (0.03 MB PDF) [file ppat.1000597.s002.pdf]

	Tc.C1	Tc.C10	Tc.B2	Tc.A6	Tc.D1	Tc.C2	Tc.E9	Tc.C3	Tc.E6	Tc.2
Tc.C1	-	14	17	11	94	74	88	107	78	103
Tc.C10	24	-	9	15	85	103	93	88	101	88
Tc.B2	12	16	-	12	93	94	102	88	83	94
Tc.A6	11	24	13	-	106	89	95	96	75	96
Tc.D1	102	92	101	96	-	22	14	102	76	92
Tc.C2	96	103	98	102	17	-	24	94	89	103
Tc.E9	88	95	79	89	20	11	-	78	103	96
Tc.C3	78	84	93	93	83	104	82	-	24	87
Tc.E6	77	102	91	89	101	87	95	17	-	101
Tc.2	99	97	97	94	102	98	103	98	91	-
