# Supplementary figures and images for: Intraspecific Epitopic Variation in a Carbohydrate Antigen Exposed on the Surface of Trichostrongylus colubriformis Infective L3 Larvae
Source: PLoS Pathog. 2009 Sep 25;5(9):e1000597. doi: 10.1371/journal.ppat.1000597 (PMC2742895; doi:10.1371/journal.ppat.1000597)

T. co  
T. ci  
C. el  
O. os  
M  
C. cu  
P. tr  
H. co  
N. sp

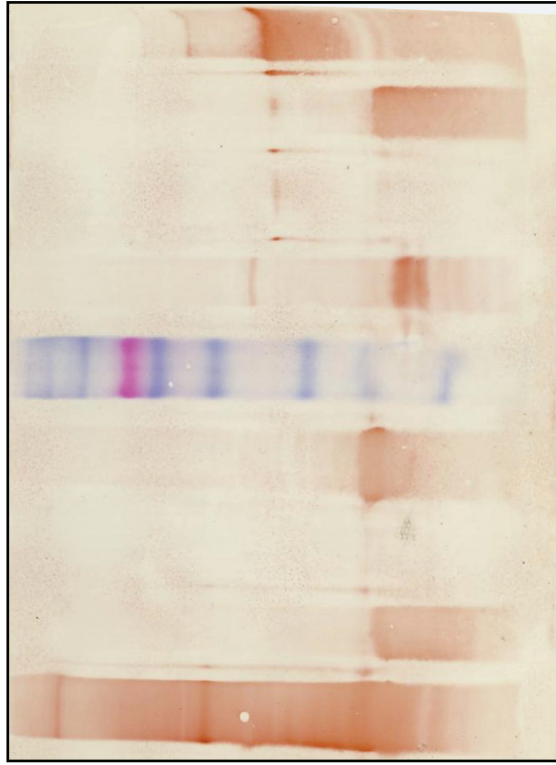

150  
100  
75  
50  
37  
25  
20  
15

Supplement: Figure S2 — Lack of species specificity of Tc.2 scFv recognizing a strongylid sheath antigen-loading control for Figure 2. Western blot was performed containing hot water extracts of L3 stage larvae prepared from eight different species of nematode. Species used were Trichostrongylus colubriformis (T. co), Teladorsagia circumcincta (T. ci), Caenorhabditis elegans (C. el), Ostertagia ostergia (O. os), Cooperia curticei (C. cu), Parastrongyloides trichosuri (P. tr), Haemonchus contortus (H. co), and Nematodirus spatiger (N. sp). The Tc.2 scFv [12] was incubated with the blots and bound scFv was detected using an anti-E-tag/HRP conjugated mAb. Markers (M) indicate the molecular weight mobility shown on left. (0.13 MB PDF) [file ppat.1000597.s003.pdf]

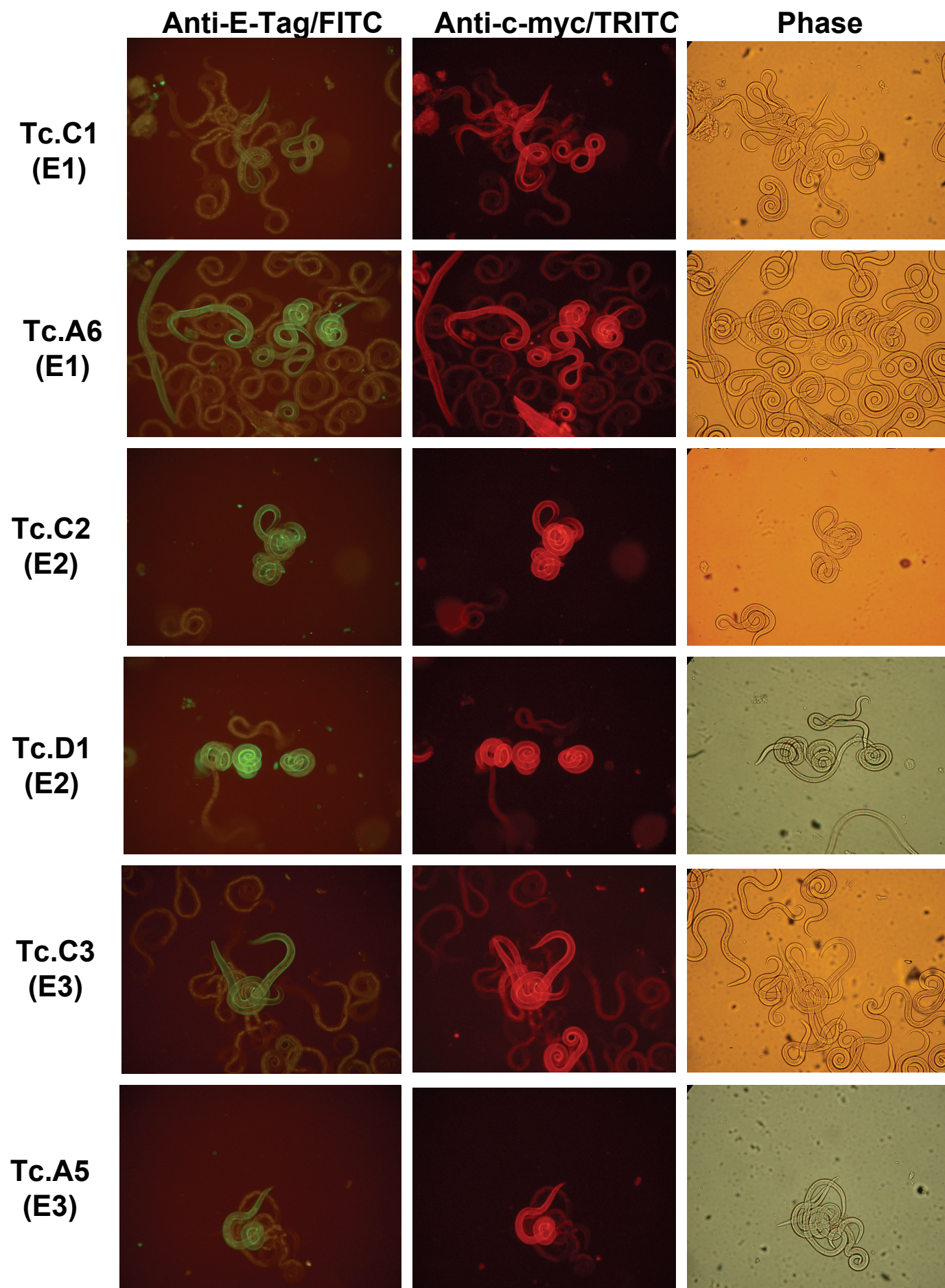

Supplement: Figure S4 — Double-staining immunofluorescence of living T. colubriformis L3 larvae probed with the same anti-CarLA scFvs fused to different epitope tags. Live T. colubriformis exsheathed L3 larvae were incubated with pools containing the same anti-CarLA scFv (as indicated) fused to two different tags, E-tag or the c-myc. After washing, bound scFv was stained with both anti-E-tag/FITC mAb and anti-c-myc/TRITC mAb and detected by fluorescence microscopy using filters optimized for FITC (green) or TRITC (red) detection. Within each group the same fields were visualized for FITC (left) and TRITC (center) or by phase contrast (right). (3.08 MB PDF) [file ppat.1000597.s005.pdf]

**Tc.D1/FITC (E2)**

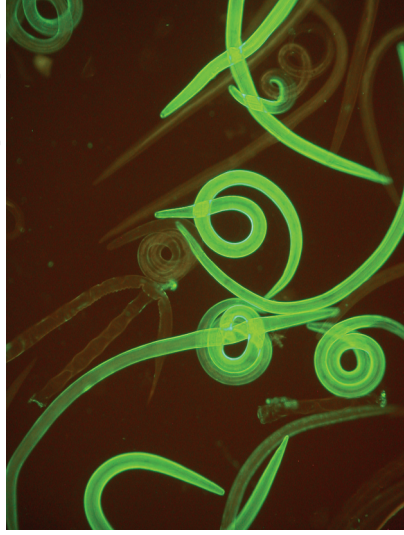

**Tc.C3/TRITC (E3)**

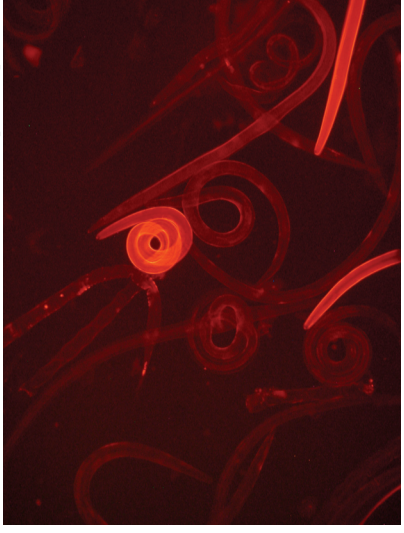

**Phase**

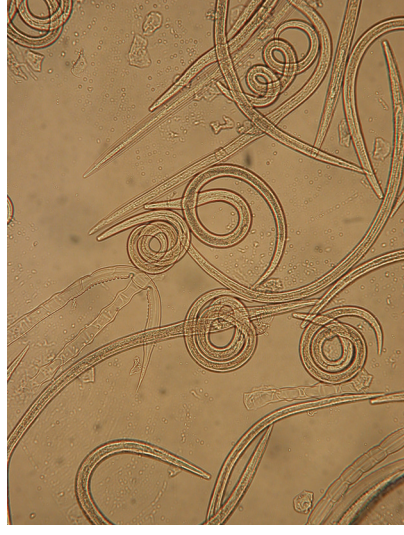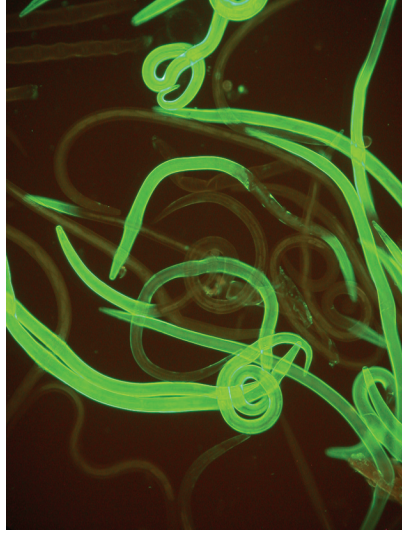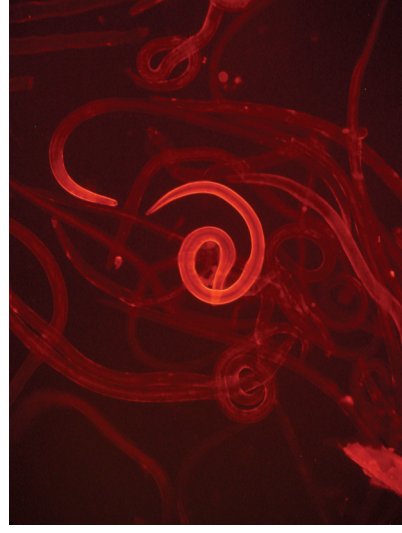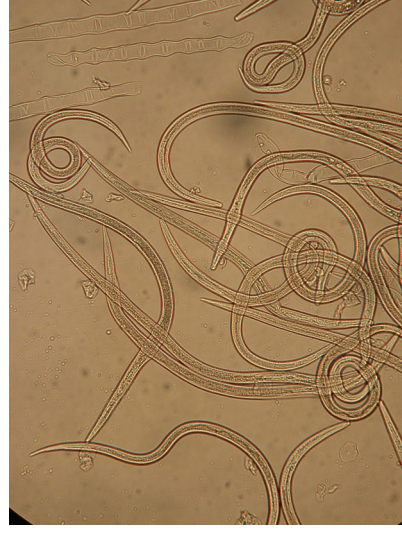

**A**

**B**

Supplement: Figure S5 — Double-staining immunofluorescence of living T. colubriformis L3 larvae probed with two anti-CarLA scFvs using sequential or pooled scFv incubations. A. Live T. colubriformis exsheathed L3 larvae were incubated with anti-CarLA scFv Tc.D1, washed, and then incubated with scFv Tc.C3. B. Live T. colubriformis exsheathed L3 larvae were incubated with a pool containing anti-CarLA scFvs Tc.D1 and Tc.C3. After washing, bound scFv was stained with both anti-E-tag/FITC mAb and anti-c-myc/TRITC mAb and detected by fluorescence microscopy using filters optimized for FITC (green) or TRITC (red) detection. Within each group the same fields were visualized for FITC (left) and TRITC (center) or by phase contrast (right). The epitope group of the anti-CarLA scFvs are indicated in parentheses. (2.47 MB PDF) [file ppat.1000597.s006.pdf]

Tc.D1/FITC (E2)

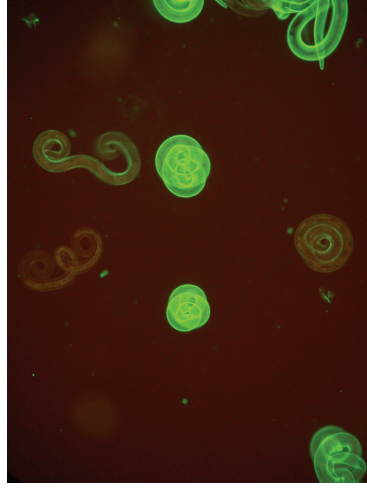

Tc.A6/TRITC (E1)

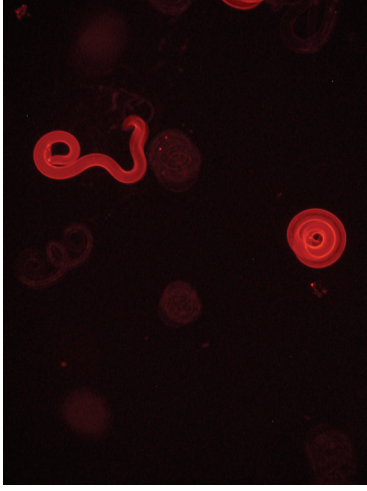

Phase

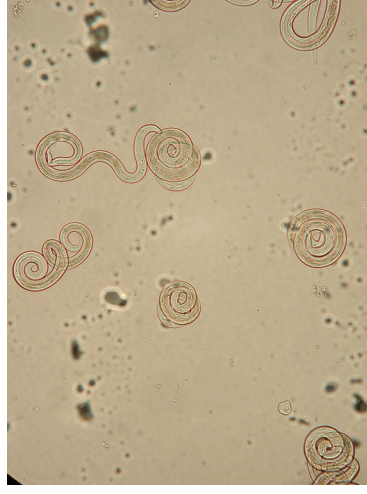

Tc.D1/FITC (E2)

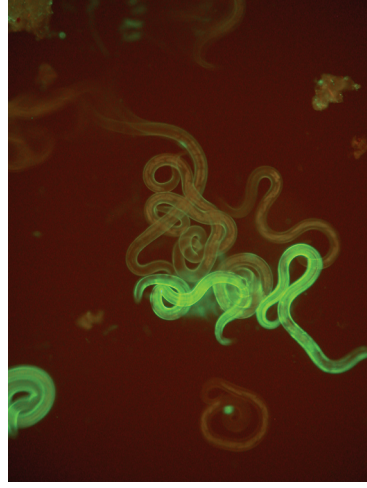

Tc.C1/TRITC (E1)

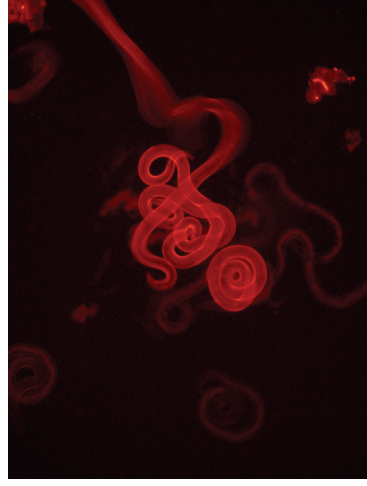

Phase

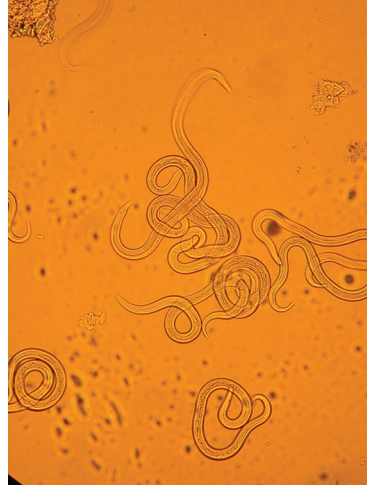

Tc.C1/FITC (E1)

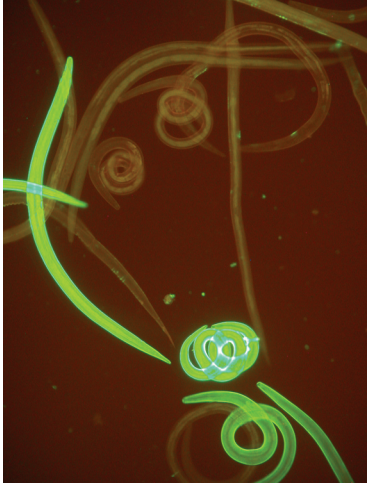

Tc.D1/TRITC (E2)

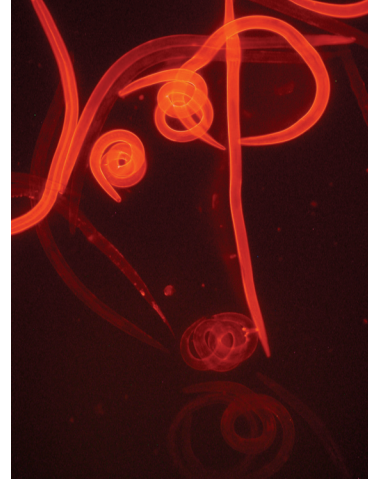

Phase

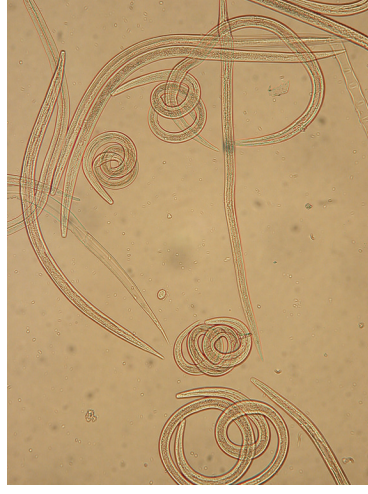

A

B

C

Supplement: Figure S6 — Double-staining immunofluorescence of living T. colubriformis L3 larvae probed with two anti-CarLA scFvs in various epitope group combinations. Live T. colubriformis exsheathed L3 larvae were incubated with pools of two anti-CarLA scFvs, each recognizing a different epitope and fused to either the E-tag or the c-myc epitopic tag. After washing, bound scFv was stained with both anti-E-tag/FITC mAb and anti-c-myc/TRITC mAb and detected by fluorescence microscopy using filters optimized for FITC (green) or TRITC (red) detection. Within each group (A-C) the same fields are visualized for FITC (left) and TRITC (center) or by phase contrast (right). A. The scFv pool was Tc.D1/E-Tag and Tc.A6/c-myc. B. The scFv pool was Tc.D1/E-Tag and Tc.C1/c-myc. C. The scFv pool was Tc.C1/E-Tag and Tc.D1/c-myc. The epitope group of the anti-CarLA scFvs are indicated in parentheses. (2.34 MB PDF) [file ppat.1000597.s007.pdf]

**Tc.A5/FITC (E3)**

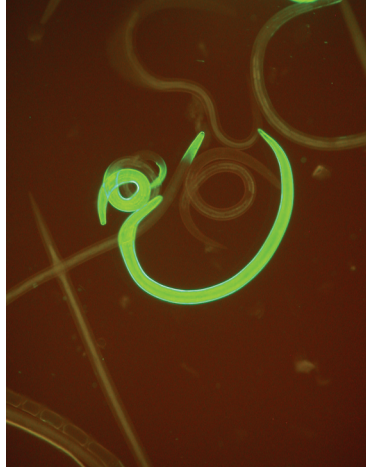

**A**

**Tc.C1/TRITC (E1)**

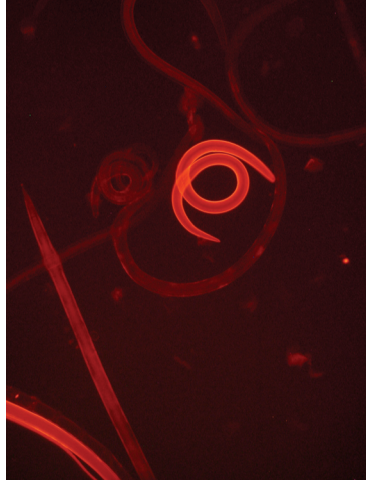

**Phase**

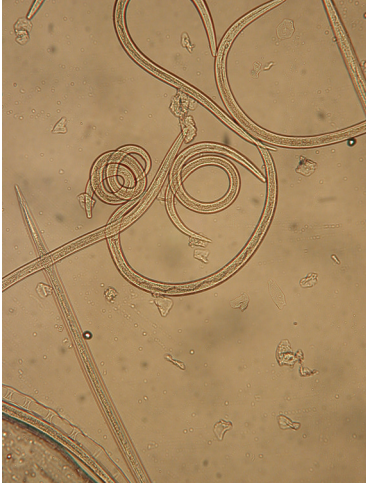

**Tc.C3/FITC (E3)**

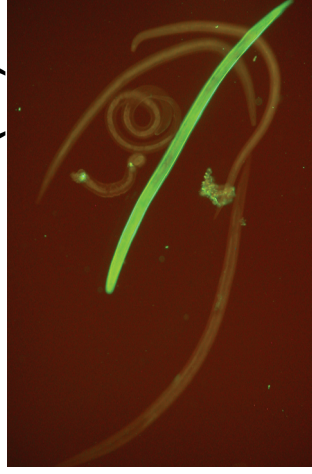

**Tc.C1/TRITC (E1)**

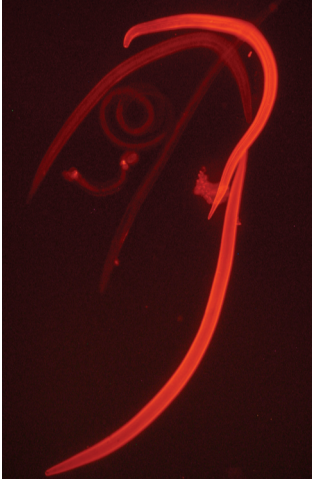

**Phase**

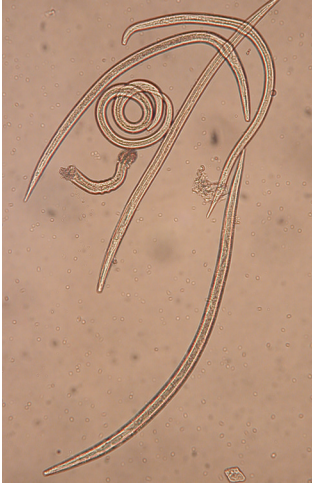

**B**

**Tc.C3/FITC (E3)**

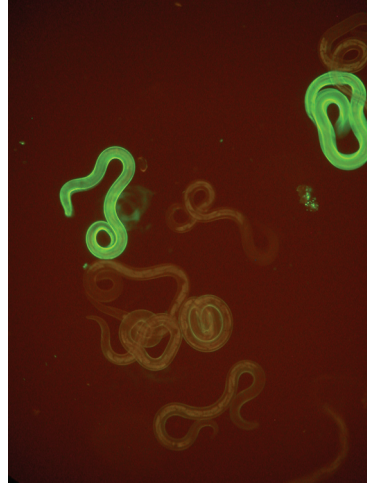

**Tc.C2/TRITC (E2)**

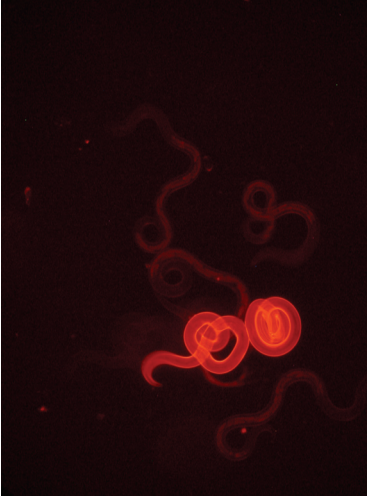

**Phase**

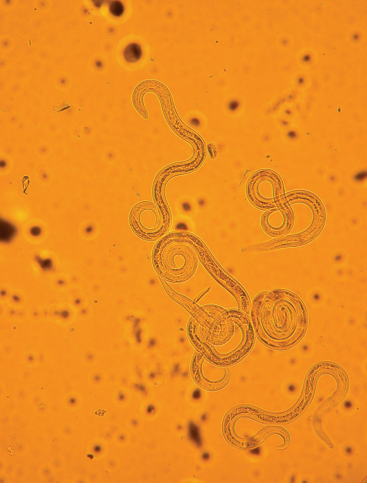

**C**

Supplement: Figure S7 — Double-staining immunofluorescence of living T. colubriformis L3 larvae probed with two anti-CarLA scFvs in various epitope group combinations. Live T. colubriformis exsheathed L3 larvae were incubated with pools of two anti-CarLA scFvs, each recognizing a different epitope and fused to either the E-tag or the c-myc epitopic tag. After washing, bound scFv was stained with both anti-E-tag/FITC mAb and anti-c-myc/TRITC mAb and detected by fluorescence microscopy using filters optimized for FITC (green) or TRITC (red) detection. Within each group (A–C) the same fields are visualized for FITC (left) and TRITC (center) or by phase contrast (right). A. The scFv pool was Tc.A5/E-Tag and Tc.C1/c-myc. B. The scFv pool was Tc.C3/E-Tag and Tc.C1/c-myc. C. The scFv pool was Tc.C3/E-Tag and Tc.C2/c-myc. The epitope group of the anti-CarLA scFvs are indicated in parentheses. (2.27 MB PDF) [file ppat.1000597.s008.pdf]
