# Supplementary material for: Intraspecific Epitopic Variation in a Carbohydrate Antigen Exposed on the Surface of Trichostrongylus colubriformis Infective L3 Larvae
Source: PLoS Pathog. 2009 Sep 25;5(9):e1000597. doi: 10.1371/journal.ppat.1000597 (PMC2742895; doi:10.1371/journal.ppat.1000597)
Supplement: Figure S3 — Sequence analysis of selected ovine scFvs. The amino acid sequences of the nine selected anti-CarLA scFvs are presented in single letter amino acid code. Alignment of the sequences was performed using DS-Gene software (Accelrys Gene 2.0). Darker shading indicates more conservation of amino acid sequence at that position. (0.31 MB PDF) [file ppat.1000597.s004.pdf]

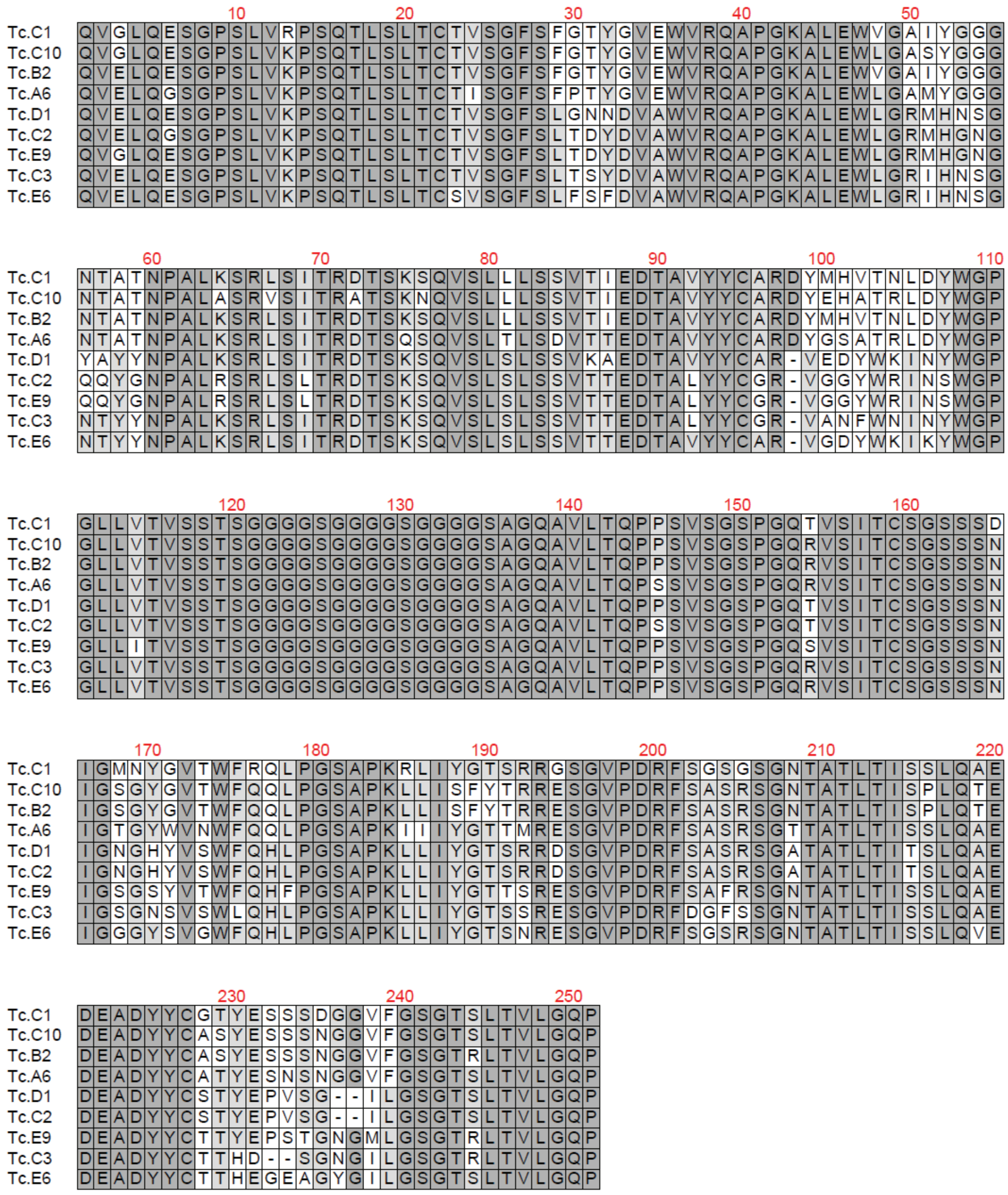
10 20 30 40 50
Tc.C1 QVGLQESGPSLVRPSQTLSLTCTVSGFSFGTYGVEWVRQAPGKALEWVGAIYGGG
Tc.C10 QVGLQESGPSLVKPSQTLSLTCTVSGFSFGTYGVEWVRQAPGKALEWLGASYGGG
Tc.B2 QVELQESGPSLVKPSQTLSLTCTVSGFSFGTYGVEWVRQAPGKALEWVGAIYGGG
Tc.A6 QVELQGSGPSLVKPSQTLSLTCTISGFSFPTYGVEWVRQAPGKALEWLGAMYGGG
Tc.D1 QVELQESGPSLVKPSQTLSLTCTVSGFSLGNNDVAWVRQAPGKALEWLGRMHNSG
Tc.C2 QVELQGSGPSLVKPSQTLSLTCTVSGFSLTDYDVAWVRQAPGKALEWLGRMHGNG
Tc.E9 QVGLQESGPSLVKPSQTLSLTCTVSGFSLTDYDVAWVRQAPGKALEWLGRMHGNG
Tc.C3 QVELQESGPSLVKPSQTLSLTCTVSGFSLTSYDVAWVRQAPGKALEWLGRIHNSG
Tc.E6 QVELQESGPSLVKPSQTLSLTCSVSGFSLFSFDVAWVRQAPGKALEWLGRIHNSG
60 70 80 90 100 110
Tc.C1 NTATNPALKSRLSITRDTSKSQVSLLLSSVTIEDTAVYYCARDYMHVTNLDYWGP
Tc.C10 NTATNPALASRVSITRATSKNQVSLLLSSVTIEDTAVYYCARDYEHATRLDYWGP
Tc.B2 NTATNPALKSRLSITRDTSKSQVSLLLSSVTIEDTAVYYCARDYMHVTNLDYWGP
Tc.A6 NTATNPALKSRLSITRDTSQSQVSLTLSDVTTEDTAVYYCARDYGSATRLDYWGP
Tc.D1 YAYYNPALKSRLSITRDTSKSQVSLSLSSVKAEDTAVYYCAR-VEDYWKINYWGP
Tc.C2 QQYGNPALRSRLSLTRDTSKSQVSLSLSSVTTEDTALYYCGR-VGGYWRINSWGP
Tc.E9 QQYGNPALRSRLSLTRDTSKSQVSLSLSSVTTEDTALYYCGR-VGGYWRINSWGP
Tc.C3 NTYYNPALKSRLSITRDTSKSQVSLSLSSVTTEDTALYYCGR-VANFWNINYWGP
Tc.E6 NTYYNPALKSRLSITRDTSKSQVSLSLSSVTTEDTAVYYCAR-VGDYWKIKYWGP
120 130 140 150 160
Tc.C1 GLLVTVSSTSGGGGSGGGGSGGGGSAGQAVLTQPPSVSGSPGQTVSITCSGSSSD
Tc.C10 GLLVTVSSTSGGGGSGGGGSGGGGSAGQAVLTQPPSVSGSPGQRVSITCSGSSSN
Tc.B2 GLLVTVSSTSGGGGSGGGGSGGGGSAGQAVLTQPPSVSGSPGQRVSITCSGSSSN
Tc.A6 GLLVTVSSTSGGGGSGGGGSGGGGSAGQAVLTQPSSVSGSPGQRVSITCSGSSSN
Tc.D1 GLLVTVSSTSGGGGSGGGGSGGGGSAGQAVLTQPPSVSGSPGQTVSITCSGSSSN
Tc.C2 GLLVTVSSTSGGGGSGGGGSGGGGSAGQAVLTQPSSVSGSPGQTVSITCSGSSSN
Tc.E9 GLLITVSSTSGGGGSGGGGSGGGGSAGQAVLTQPPSVSGSPGQSVSITCSGSSSN
Tc.C3 GLLVTVSSTSGGGGSGGGGSGGGGSAGQAVLTQPPSVSGSPGQRVSITCSGSSSN
Tc.E6 GLLVTVSSTSGGGGSGGGGSGGGGSAGQAVLTQPPSVSGSPGQRVSITCSGSSSN
170 180 190 200 210 220
Tc.C1 IGMNYGVTWFRQLPGSAPKRLIYGTSRRGSGVPDRFSGSGSGNTATLTISSLQAE
Tc.C10 IGSGYGVTWFQQLPGSAPKLLISFYTRRESGVPDRFSASRSGNTATLTISPLQTE
Tc.B2 IGSGYGVTWFQQLPGSAPKLLISFYTRRESGVPDRFSASRSGNTATLTISPLQTE
Tc.A6 IGTGYWVNWFQQLPGSAPKIIIYGTTMRESGVPDRFSASRSGTTATLTISSLQAE
Tc.D1 IGNGHYVSWFQHLPGSAPKLLIYGTSRRDSGVPDRFSASRSGATATLTITSLQAE
Tc.C2 IGNGHYVSWFQHLPGSAPKLLIYGTSRRDSGVPDRFSASRSGATATLTITSLQAE
Tc.E9 IGSGSYVTWFQHFPGSAPKLLIYGTTSRESGVPDRFSAFRSGNTATLTISSLQAE
Tc.C3 IGSGNSVSWLQHLPGSAPKLLIYGTSSRESGVPDRFDGFSSGNTATLTISSLQAE
Tc.E6 IGGGYSVGWFQHLPGSAPKLLIYGTSNRESGVPDRFSGSRSGNTATLTISSLQVE
230 240 250
Tc.C1 DEADYYCGTYESSSDGGVFGSGTSLTVLGQP
Tc.C10 DEADYYCASYESSSNGGVFGSGTSLTVLGQP
Tc.B2 DEADYYCASYESSSNGGVFGSGTRLTVLGQP
Tc.A6 DEADYYCATYESNSNGGVFGSGTSLTVLGQP
Tc.D1 DEADYYCSTYEPVSG--ILGSGTSLTVLGQP
Tc.C2 DEADYYCSTYEPVSG--ILGSGTSLTVLGQP
Tc.E9 DEADYYCTTYEPSTGNGMLGSGTRLTVLGQP
Tc.C3 DEADYYCTTHD--SGNGILGSGTRLTVLGQP
Tc.E6 DEADYYCTTHEGEAGYGILGSGTSLTVLGQP
